# Attachment as a Developmental Lens for Understanding Neurodivergence: A Clinical-Theoretical Proposal

**DOI:** 10.3390/children12121703

**Published:** 2025-12-17

**Authors:** Vincenzo Maria Romeo

**Affiliations:** 1 Department of Culture and Society, University of Palermo, Viale Delle Scienze, Ed. 15, 90128 Palermo, Italy; vincenzomaria.romeo@unipa.it; Tel.: +39-3405803854; 2 School of Psychoanalytic and Groupanalytic Psychotherapy S.P.P.G., Via Fontana n° 1, 89131 Reggio Calabria, Italy; 3 Neurosinc, Via Paolo Bentivoglio n° 62, 95125 Catania, Italy

**Keywords:** autism spectrum disorder (ASD), attention-deficit/hyperactivity disorder (ADHD), attachment, social-cognitive processes, intersubjectivity, affect regulation, sensory processing, educational outcomes, inclusive education, developmental psychopathology

## Abstract

**Highlights:**

**What are the main findings?**

- Early attachment shapes regulation, intersubjectivity, and sensory processing.
- These systems channel distinct neurodivergent trajectories (autism, ADHD, and sensory profiles).

**What are the implications of the main findings?**

- Attachment- and sensory-informed assessment can refine diagnostic formulation.
- Therapy and classrooms should calibrate co-regulation and sensory load to sustain engagement.

**Abstract:**

The present manuscript proposes an integrative clinical-theoretical framework that explores neurodivergence through the lens of attachment theory, aiming to enrich our understanding of atypical developmental trajectories. Drawing from both classical Bowlby–Ainsworth models and contemporary contributions from developmental psychopathology and affective neuroscience, I examine how early relational experiences shape the regulation of affect, intersubjectivity, and sensory processing in neurodivergent populations. Particular focus is given to autism spectrum conditions, attention-deficit/hyperactivity disorder (ADHD), and sensory integration challenges, highlighting the interdependence between attachment patterns and core neurodevelopmental features. By reframing neurodivergence not merely as a deficit or disorder but as an expression of individual variation in neuro-affective development, the manuscript offers implications for diagnostic refinement, therapeutic alliance building, and psychoeducational planning. This interdisciplinary approach aims to foster a more inclusive and relationally attuned clinical paradigm that addresses both the strengths and vulnerabilities of neurodivergent children and adolescents.

## 1. Introduction

Across the last decade, “neurodivergence” has gained traction as an umbrella construct foregrounding variability in neuro-affective development rather than reducing difference to deficit. The neurodiversity perspective—originating in autistic self-advocacy and subsequently informing developmental science—frames conditions such as autism spectrum disorder (ASD) and attention-deficit/hyperactivity disorder (ADHD) as part of the natural human variation while acknowledging support needs and impairment when present [1,2]. This reframing aligns with contemporary dimensional views of neurodevelopment that emphasize heterogeneity, life-span continuity, and the salience of context and co-occurrence [2]. Within this broader shift, attachment theory offers a developmentally anchored, relational lens able to integrate affect regulation, intersubjectivity, and sensory processing—domains central to both neurodivergence and caregiving environments.

Attachment theory, articulated by Bowlby and operationalized by Ainsworth, posits that repeated caregiver–infant transactions organize neural and behavioral systems for proximity-seeking, safety, exploration, and affective co-regulation [3,4]. Classic constructs—secure base, safe haven, and internal working models—map onto processes frequently altered or differently calibrated in neurodivergent development. In parallel, intersubjectivity research elaborates early “self-with-other” motives and the fine-grained timing of embodied signals within dyads [5]. Work on parent–child synchrony shows that micro-level matching and repair of gaze, affect, and touch scaffold self-regulation and social communication across typical and atypical development [6]. Converging affective neuroscience links these relational processes to neurobiological systems supporting right-hemisphere-dominant regulation, arousal modulation, and social engagement [7]. Together, these traditions motivate the present proposal: to use attachment as a developmental lens for understanding neurodivergence—not as an alternative to neurobiology but as an integrative scaffold that organizes it in relational time.

The literature on attachment in autism has moved beyond early debates over whether autistic children form selective attachments. Meta-analytic and longitudinal evidence indicates that many autistic children do form organized attachments, with average security somewhat lower than in non-autistic groups and variability linked to cognitive level, language, and symptom severity [8,9]. Systematic reviews report secure classifications in roughly half of autistic samples assessed with separation–reunion paradigms, alongside elevated disorganization and associations with caregiving risk contexts [10]. These findings suggest that social communication differences do not preclude using caregivers for co-regulation but may render the enactment and reading of attachment signals more effortful and context-dependent. Moreover, across studies of parental sensitivity, observed sensitivity can be broadly comparable between groups, yet the strength of association between sensitivity and security varies with child characteristics (e.g., social reciprocity, sensory responsivity), underscoring the value of models that explicitly incorporate neurodivergent sensory-affective profiles into attachment processes [8,9,10].

Attachment is also implicated in attentional self-regulation. Relative to typically developing peers, children with ADHD show higher rates of insecure (notably ambivalent/resistant) and disorganized attachment representations, even when accounting for family context; such representations may also moderate response to parenting interventions [11]. A quantitative synthesis further shows that insecure—and especially disorganized—attachment is modestly but reliably associated with children’s attention problems, indicating transdiagnostic links between early relational security and attentional control trajectories [12]. Rather than implying simple causal pathways from attachment to ADHD, these data support bidirectional influences: temperamental and neurocognitive liabilities challenge dyadic regulation, while relational histories shape the development and deployment of attentional and emotion-regulation strategies.

A third pillar—sensory processing—has often been siloed despite its clear relevance to both ASD and ADHD. The occupational therapy literature proposes taxonomies of sensory processing differences (e.g., modulation, discrimination, sensory-based motor patterns) that, while debated, have catalyzed measurement and intervention work [13]. Sensory features (over- and under-responsivity, or seeking) are highly prevalent in autism and demonstrably shape social participation, learning, and stress physiology [14,15]. Yet these profiles unfold in relational contexts: synchrony, touch, and prosody deliver graded sensory input that can either amplify or buffer arousal, with cascading effects on attention and engagement. From this vantage, sensory processing is not merely an intrinsic trait but a property of dyadic systems operating under variable environmental loads, clarifying why the same caregiving behavior is regulating for one child and dysregulating for another, and why “good-enough” sensitivity sometimes appears less predictive of security in neurodivergent dyads.

Despite these convergences, current models remain fragmented. Autism research often treats attachment as a consequence or an outcome rather than a mechanism embedded in neuro-affective development; ADHD research links attachment to self-regulation but rarely engages intersubjective micro-processes; and sensory science advances typologies without consistently theorizing their relational embedding. Conceptual ambiguity further complicates translation, including conflations of attachment classifications with “attachment disorders” and imprecise terminology across research and practice [16]. Recent theoretical work also highlights the coexistence of multiple “attachment discourses” across disciplines, hampering integrative synthesis and clinical decision-making [17]. The field therefore lacks a coherent framework that simultaneously (a) honors neurodivergent strengths and liabilities; (b) situates affect regulation, intersubjectivity, and sensory processing within caregiver–child systems; and (c) yields operational implications for assessment and intervention.

This article addresses that gap by advancing an integrative clinical-theoretical proposal: “Attachment as a Developmental Lens for Neurodivergence.” Building on Bowlby–Ainsworth foundations and contemporary contributions from intersubjectivity research, synchrony science, and affective neuroscience, I articulate testable propositions about how attachment-related processes—secure base/safe haven use, dyadic repair, and co-regulatory scaffolding—interact with neurodivergent profiles in ASD, ADHD, and sensory integration challenges. I frame neurodivergence as a neuro-affective developmental variant shaped by the fit between child characteristics and caregiving affordances, rather than a fixed deficit. Clinically, I derive implications for case formulation (e.g., mapping dyadic regulatory loops and sensory affordances), therapeutic alliance building (e.g., optimizing the “felt safety” and sensory ecology of sessions), and psychoeducational planning (e.g., aligning school accommodations with attachment-informed co-regulation and sensory needs). Finally, I outline measurement and research directions—multi-level, dyadic, and developmentally sensitive—aimed at a relationally attuned, inclusive paradigm that recognizes both strengths and vulnerabilities in neurodivergent children and adolescents.

Autism Spectrum Disorder (ASD). ASD is a neurodevelopmental condition characterized by differences in social communication and interaction alongside restricted and repetitive patterns of behavior, interests, or sensory processing. Phenotypic expression is highly heterogeneous, with frequent co-occurring conditions and variable support needs across development. Attention-deficit/hyperactivity disorder (ADHD) is a neurodevelopmental condition featuring developmentally inappropriate levels of inattention and/or hyperactivity and impulsivity, underpinned by alterations in attentional control, inhibitory processes, delay aversion, and motivational salience. It is dimensional in expression and often persists, at least in part, into adolescence and adulthood.

Terminology note: For clarity and consistency, I use ASD throughout when referring to diagnostic/nosological constructs; when discussing the broader neurodiversity discourse, I clarify explicitly if “autism spectrum conditions (ASCs)” are intended.

This manuscript is a perspective/concept paper. I advance a testable, bidirectional framework that organizes affect regulation, intersubjectivity, and sensory processing within caregiver–child systems and translates directly to clinical and educational practice, rather than reporting new empirical data. Unlike prior accounts that treat attachment and sensory processing as parallel or independent streams, I propose an integrated model that specifies mechanisms, predicts conditional effects, and yields actionable levers for assessment, intervention, and school collaboration.

## 2. Theoretical Background

### 2.1. Attachment Theory

Attachment theory, originating in Bowlby’s ethological/control systems account and Ainsworth’s naturalistic observations, describes how early caregiving organizes regulatory, relational, and representational systems across development. Contemporary syntheses depict attachment as a probabilistic, canalized system rather than a fixed typology and emphasize dimensional individual differences (e.g., security vs. insecurity indexed by anxiety/avoidance) that show modest-to-moderate stability alongside context-dependent change [18]. Lifespan analyses further indicate that selection and socialization jointly account for continuity and change in attachment representations, arguing against determinism and highlighting malleability with environmental inputs [19].

Converging affective and social neuroscience anchor attachment in biobehavioral mechanisms. Cross-species and human work delineates coordinated neuroendocrine, autonomic, and cortico–subcortical systems supporting proximity seeking, stress attenuation, and dyadic co-regulation—embedding attachment within neuroaffective regulatory networks that scaffold socioemotional development [20]. These accounts dovetail with mentalizing models that link early attachment experiences and caregiver sensitivity to a multi-level neurocognitive system (default-mode, salience, control networks) for representing self/other mental states under affective load [21].

Critical refinement concerns the taxonomy of insecurity and the status of disorganization. Insecure patterns (avoidant, resistant) are coherent, strategy-based adaptations, whereas disorganized attachment denotes a breakdown of strategy under stress and should neither be equated with maltreatment per se nor confused with “attachment disorder.” Consensus reviews caution against forensic over-interpretation, stress heterogeneity, and contextual malleability, and document intervention responsiveness [22]. Programmatic appraisals call for conceptual clarity and methodological pluralism: distinguishing trait-like representational measures from state-like dyadic processes; integrating behavioral, physiological, and neural indicators; and pivoting from categorical debates to cross-cutting processes such as regulation, synchrony, and intersubjectivity [23].

In sum, modern attachment science offers (i) dimensional, developmentally sensitive constructs; (ii) multi-systemic mechanisms (neuroendocrine, autonomic, and corticolimbic); and (iii) clinically relevant distinctions (e.g., disorganization vs. diagnostic entities). These facets provide a robust substrate for interfacing with neurodevelopmental conditions in which regulatory load, social signal processing, and sensory responsivity are central.

### 2.2. Neurodivergence

Neurodivergence denotes naturally occurring variability in neurocognitive development, yielding distinct profiles of perception, attention, and affect regulation. In clinical nosology, major categories include autism spectrum disorder (ASD) and attention-deficit/hyperactivity disorder (ADHD), alongside sensory processing differences frequently documented in ASD and observed dimensionally across presentations [24]. Authoritative overviews characterize ASD by differences in social communication and restricted/repetitive patterns with high heterogeneity, frequent co-occurrence, and multifactorial etiopathogenesis comprising polygenic architectures and convergent developmental pathways [24]. ADHD is likewise polygenic and heterogeneous, with dysregulation of attention, inhibitory control, motivational systems, and persistence into adolescence/adulthood forming a substantial subset [25].

A deficit-only model obscures heterogeneity and strengths (e.g., monotropism-linked deep focus, systemizing talents, creativity, resilience). The neurodiversity perspective reframes atypicalities as differences with both adaptive potential and disability-related needs contingent on person–environment fit, advocating participatory science, ecological validity, and outcome metrics that prioritize quality of life alongside impairment reduction [26]. Programmatic proposals call for “shifting the normal science” of autism toward co-produced agendas, multi-level mechanisms linked to lived experience, and translational pipelines that reduce health/disability inequities [27].

Sensory integration has particular salience across neurodivergent profiles: in ASD, sensory hyper-/hyporesponsivity and atypical multisensory integration are core and shape social attention, learning, and emotion regulation. Integrative neuroscience synthesizes symptom- and circuit-level evidence—altered habituation, predictive-coding precision, and thalamocortical gating— that can amplify environmental load, intensify avoidance, or yield “challenging” behaviors secondarily to distress rather than volitional oppositionality [26]. Within a developmental–relational frame, these sensory differences interact with caregiving ecologies (e.g., pacing, predictability, and graded novelty) and broader contexts (e.g., classroom load) to produce developmental cascades.

Positioning neurodivergence in this way encourages clinicians and researchers to (i) specify both strengths and vulnerabilities; (ii) model contextual amplification or buffering; and (iii) design supports that target not only skills but also environments (e.g., sensory accommodations, co-regulatory routines, alliance-centered care). This orientation dovetails with attachment-informed constructs of sensitivity, synchrony, and dyadic regulation and motivates an integrative lens.

### 2.3. Intersection of Attachment–Neurodivergence

Empirical work increasingly interrogates how attachment processes intersect with neurodivergent phenotypes. In ASD, systematic review evidence indicates that many autistic children form selective, organized attachments; security is present in a substantial proportion, with variability associated with caregiver sensitivity, cognitive level, and symptom severity rather than diagnosis [28]. A meta-analysis further shows that maternal sensitivity relates to child attachment security in ASD with effect sizes comparable to non-autistic populations, suggesting conserved caregiving pathways despite phenotypic differences [29]. In ADHD, a systematic review documents elevated insecure attachment and associations between attachment insecurity, symptom severity, and emotion dysregulation, while calling for longitudinal, mechanistic designs [30]. Across child samples more broadly, meta-analytic evidence links insecure attachment with attention problems, underscoring transdiagnostic relevance of early relational security for self-regulation trajectories [31].

Proposed mechanisms converge on three overlapping systems. 1. Affect regulation/stress physiology: Secure-base caregiving supports allostatic regulation and scaffolds top-down control, offering compensatory resources for children with higher regulatory demand—an inference consistent with dyadic regulation models synthesized in developmental neuroscience (see Section 2.1) and elaborated with second-person approaches below. 2. Social orienting/intersubjectivity: Dyadic synchrony—moment-to-moment coordination of attention, affect, and physiology—facilitates joint engagement and mentalizing; hyperscanning in early mother–infant interactions, including elevated-likelihood-of-autism cohorts, documents inter-brain synchrony as a plausible mechanism linking caregiving signals to social–cognitive outcomes [32]. 3. Sensory processing/gating: Sensory over-responsivity (SOR) plausibly links environmental load to reduced social availability and co-regulation; neuroimaging demonstrates sensorilimbic hyper-responsivity and thalamocortical dysmodulation in autistic youth with SOR, aligning atypical gating with distress and avoidance that can be buffered by graded co-regulation and environmental adaptation [33,34].

Clinically, these findings argue against pathologizing autism or ADHD as failures of attachment and instead support bidirectional models: neurodivergent dispositions shape how children signal needs and process input; caregiver sensitivity, predictability, and repair shape how those dispositions are regulated and expressed. Attachment-informed, neurodiversity-affirming care emphasizes (i) tuning interventions to sensory thresholds and attentional style; (ii) building caregiver co-regulatory and mentalizing capacities under stress; and (iii) leveraging strengths (e.g., special interests, monotropism, and novelty-seeking) to recruit engagement. Second-person studies show that modulating adult gaze increases information coupling between infant and adult brains, illustrating a micro-mechanism by which attuned signaling may foster engagement [35]. More broadly, the synchrony literature delineates micro-processes of matching and repair that structure self-regulation and participation across typical and atypical development, offering actionable targets for assessment and intervention [36].

## 3. Proposed Integrative Framework

**Figure 1:**
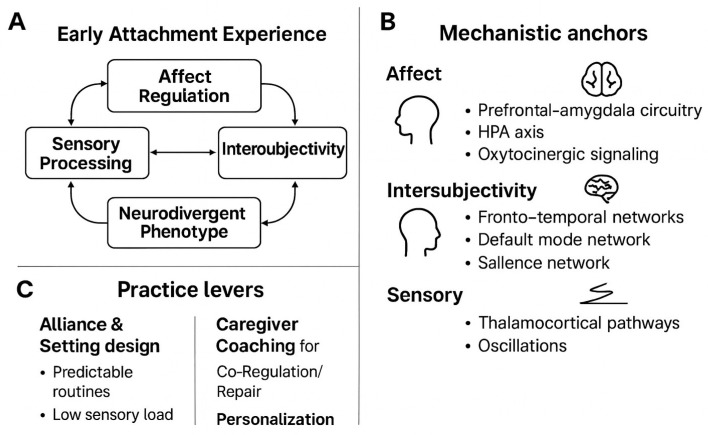
Attachment-informed integrative framework linking early attachment experiences to affect regulation, intersubjectivity, sensory processing, and neurodivergent phenotypes. **Legend:** Attachment-informed integrative framework (multi-panel). (**A**) Core schema: Early attachment experience influences three partially overlapping systems—affect regulation, intersubjectivity, and sensory processing—that channel trajectories toward neurodivergent phenotypes; double-headed arrows indicate bidirectional coupling. (**B**) Mechanistic anchors: Stress buffering and prefrontal–amygdala control (affect), fronto-temporal networks supporting synchrony and mentalizing (intersubjectivity), and thalamocortical/oscillatory gating (sensory); oxytocinergic pathways support social buffering. (**C**) Practice levers: Alliance and setting design (predictability and low sensory load), caregiver coaching for co-regulation/repair, and personalization by susceptibility. The model predicts moderated mediation: attachment-related processes improve outcomes via affective, intersubjective, and sensory pathways, with effect sizes contingent on susceptibility and reciprocal influences from neurodivergent traits.

I propose a multi-level, attachment-informed framework in which early attachment experiences organize three partially overlapping regulatory systems—affect regulation, intersubjectivity, and sensory processing—that, in turn, channel developmental trajectories toward distinct neurodivergent phenotypes (Figure 1). The model is explicitly transactional and bidirectional: child neurocognitive dispositions (e.g., attentional control, arousal thresholds, and sensory responsivity) co-determine the efficiency of dyadic co-regulation, which subsequently recalibrates those very dispositions across time and contexts [37].

What is new in this framework?

- Integration: A single, bidirectional model linking attachment, intersubjectivity, and sensory processing to neurodivergent trajectories.
- Multi-level timing: Microtemporal (repair), developmental (capacity consolidation), and contextual (sensory ecology) levels with cross-level interactions.
- Mechanistic specificity: Clear affective, intersubjective, and sensory pathways that can be falsified with feasible assays.
- Sensory ecology as first-order determinant of relational availability and co-regulation in everyday settings.
- Personalization: Differential/vantage sensitivity predicts who benefits most and under what conditions.

### 3.1. Model Presentation

**Nodes and links.** The framework comprises five nodes: **early attachment experience** (caregiver sensitivity/contingency, repair, secure base/safe haven), **affect regulation** (allostatic-load management, stress reactivity and recovery), **intersubjectivity** (social orienting, joint attention, mentalizing under affective arousal), **sensory processing** (thresholds, gating, multisensory integration), and the **neurodivergent phenotype** (behaviorally expressed profiles across autism spectrum conditions, ADHD, and prominent sensory differences). In autism, variability in attachment security appears linked less to diagnosis per se than to child characteristics and caregiving contingencies, indicating conserved pathways of sensitivity–security coupling [38]. In ADHD, attachment representations show higher rates of insecurity relative to typically developing peers and relate to symptom severity and emotion dysregulation, supporting developmental models that integrate caregiving with self-regulation [39]. Beyond disorder labels, meta-analytic evidence connects insecure attachment with attention problems across child samples, underscoring transdiagnostic relevance of early relational security for attentional control trajectories [40].

**Sensory pathways.** Neurobiological studies identify sensorilimbic hyper-responsivity as a correlate of SOR in autistic youth, pointing to mechanisms through which environmental load can drain social availability and undermine co-regulation [41]. Complementary work documents thalamocortical dysmodulation during sensory stimulation, suggesting that atypical gating at early relay stages may propagate to higher-order networks crucial for engagement and learning [42].

**Dyadic process anchor.** Research on parent–infant synchrony details how micro-level matching and iterative repair in gaze, affect, touch, and physiology scaffold joint engagement and self-regulation across development, situating attachment within measurable dyadic processes that can be targeted for change [43].

**Levels of influence.** I delineate three analytic levels: **(i) microtemporal** (seconds–minutes; e.g., perturbation and repair), **(ii) developmental** (months–years; e.g., consolidation of executive control and internal working models), and **(iii) contextual** (family, school, and clinical settings that amplify or buffer regulatory load). Each level affords concrete measurement targets—behavioral micro-coding, autonomic/neuroendocrine indices, and naturalistic classroom observation—and is expected to exhibit **cross-level interactions** (e.g., microtemporal synchrony forecasting longitudinal gains in joint attention under manageable sensory load).

**Testable propositions.**

**H1 (Affect).**  *Higher caregiver contingency predicts faster stress recovery and stronger top-down control; clinically, this guides co-regulation targets and HRV-informed titration [44,45,46].*

**H2 (Intersubjectivity).**  *Greater dyadic synchrony improves joint engagement and mentalizing; educationally, teachable micro-skills (gaze, prosody, and immediate repair) increase participation [47,48].*

**H3 (Sensory).**  *Sensory-aware co-regulation reduces SOR-related reactivity; in practice, predictable routines and graded novelty sustain alliance and learning [49,50,51].*

**H4 (Differential benefits).**  *Children high in environmental susceptibility show larger gains under optimized caregiving and sensory ecologies; personalize dosage and monitor responders [52,53].*

### 3.2. From Attachment to Regulatory Systems

**Affect regulation.** Sensitive, predictable caregiving scaffolds allostatic regulation by dampening excessive threat appraisal and by supporting prefrontal modulation of limbic salience; in children with elevated baseline arousal or attentional control challenges, this dyadic buffering should shorten recovery time constants after perturbations and reduce spillover of arousal into cognition and behavior.

**Intersubjectivity.** Attachment processes organize self-with-other timing and meaning-making. Moment-to-moment synchrony coordinates attention, affect, and physiology, thereby facilitating joint attention and mentalizing under affective load; targeted manipulation of ostensive cues (e.g., caregiver gaze) provides causal leverage on these processes within second-person paradigms already shown to modify neural coupling.

**Sensory processing.** Attachment unfolds within a sensory ecology. Predictive-processing accounts of autism posit aberrant precision, i.e., atypically high confidence assigned to sensory prediction errors, making contexts feel “too real” and heightening the costs of proximity and novelty [54]. Converging formulations propose that altered precision of prediction across hierarchical levels can distort sensory gating and increase regulatory load, rendering graded, predictable input as a primary route to stabilization [55]. An attachment-informed titration of sensory input—paced proximity, predictable transitions, graded novelty—should therefore reduce uncertainty, sustain engagement, and preserve bandwidth for learning.

### 3.3. Bidirectionality: Neurodivergent Traits Shaping Attachment Processes

**The framework rejects unidirectional causality.** Neurodivergent dispositions alter how attachment signals are expressed, read, and used: fluctuations in attention and hyper-arousal can transiently reduce visible proximity-seeking—not because the caregiver lacks value, but because the approach entails excessive sensory/attentional demand in the moment. Monotropism—a disposition toward deep, narrow interests—may constrain opportunities for joint attention yet offers high-motivation channels for engagement when leveraged therapeutically within the alliance and daily routines [56]. Such child-driven parameters can partially decouple classic sensitivity–security associations and moderate responses to relationship-based and sensory-aware interventions.

### 3.4. Clinical Relevance: Attachment-Informed, Neurodiversity-Affirming Practice

The model yields three families of clinical levers.

(1) **Alliance and setting design (felt safety).** Optimize the sensory ecology of encounters: predictable routines, low-load auditory environments, modulated lighting, graded proximity; incorporate co-regulating stimuli and interest-based materials to recruit approach without overshooting thresholds.
(2) **Caregiver coaching and co-regulation**. Train caregivers in contingency mapping, paced responsiveness, and repair strategies; embed these skills in attachment-based and parent-mediated programs (e.g., ABC; PACT) adapted to each child’s sensory thresholds and attentional style.
(3) **Personalization by susceptibility**. Because children differ in environmental susceptibility/vantage sensitivity, those at the high-sensitivity tail should show larger gains with enriched, sensory-aware scaffolding, while chaotic settings may be disproportionately harmful. This provides a principled basis for precision support in which dosage and delivery are indexed to susceptibility markers (e.g., SOR severity, autonomic flexibility, and stress reactivity).

### 3.5. Measurement and Research Program

A multi-method agenda can adjudicate mechanisms: microtemporal assays (micro-coded synchrony; brief perturbation/repair; wearable autonomic sensors) to quantify contingency dynamics; second-person neuroscience (dual-EEG/fNIRS) to capture inter-brain coupling during manipulated ostensive cues; sensory load titration pairing psychophysics with connectivity analysis to test SOR mediation; and moderation designs to identify for whom and under what conditions attachment-informed supports maximize participation and learning. Pragmatic trials can embed sensory-aware co-regulation within parent- and attachment-based interventions to evaluate cascading effects on school inclusion and well-being.

## 4. Clinical Implications

Translating the framework into practice requires integrated assessment, attachment- and sensory-attuned psychotherapy, and education/training for caregivers and teachers. Evidence on SOR and thalamocortical dysmodulation in autism underscores why sensory context is a first-order determinant of relational availability and co-regulation [57,58]. Individual differences in environmental susceptibility and vantage sensitivity further argue for personalized supports, dosing, and careful titration of arousal [59,60,61]. Trial work indicates that sensory-integration protocols, attachment-based interventions, and parent-mediated communication programs represent feasible levers when adapted to the child’s thresholds and dyadic needs [62,63,64]. These strands converge on a pragmatic roadmap that links mechanisms to clinical and school outcomes.

### 4.1. Diagnosis: Integrating Relational and Sensory Assessment

A mechanism-informed formulation should pair gold-standard neurodevelopmental tools with attachment/mentalizing and sensory measures and—where feasible—physiological indices of regulation.

**Limitations of Current Diagnostic Systems and Rationale for an Attachment-Informed Approach.** Categorical nosologies such as DSM/ICD capture symptom clusters but are strained by marked heterogeneity, extensive co-occurrence, and limited attention to relational processes and sensory profiles. Categorical thresholds may obscure dimensional variation that is clinically decisive (e.g., sensory load and co-regulation needs), invite “symptom overshadowing”, and predict treatment response only weakly. An attachment-informed formulation complements diagnostic criteria by mapping dyadic contingency/repair, caregiver reflective functioning, and sensory thresholds that jointly shape stress reactivity, participation, and learning. Brief observational tools (e.g., emotional availability—EA), caregiver mentalizing measures, and structured sensory profiling, optionally paired with simple physiological indices (e.g., HRV), add mechanistic resolution to standard assessments. In this perspective, diagnosis is refined by specifying how child characteristics and caregiving affordances interact to produce the observed phenotype and by identifying leverage points for intervention.

**Relational/mentalizing assessment**. Caregiver reflective functioning is efficiently captured with the Parental Reflective Functioning Questionnaire (PRFQ) (brief self-report) or interview-based ratings, indexing the parent’s capacity to apprehend the child as a psychological agent under affective load [65]. EA observation quantifies caregiver sensitivity, structuring, non-intrusiveness/non-hostility, and the child’s responsiveness/involvement; it is feasible in brief play samples and sensitive to dyadic change [66,67].

**Neurodevelopmental phenotyping.** For ADHD, practice guidance emphasizes multi-informant rating scales, impairment across settings, and systematic assessment of co-occurring conditions—principles readily harmonized with an attachment-developmental lens [68]. For autism, standardized observation (e.g., ADOS-revised algorithms) remains foundational for differential diagnosis and phenotyping [69], while quantitative social responsivity measures (e.g., SRS/SRS-2) index traits across contexts and enables change tracking in routine care and trials [70].

**Sensory profiling and school ecology.** Standardized approaches and fidelity-anchored sensory-integration frameworks support consistent characterization and treatment planning in clinical and educational settings [71]. Because classroom load interacts with sensory thresholds, school-relevant assessment should anticipate noise/visual density/transitions; SOR has been linked to classroom emotional/behavioral and learning barriers, motivating proactive accommodation planning [72].

**Physiology and micro-processes**. When available, heart-rate variability (HRV), electrodermal activity, or stress biomarkers can index allostatic load and treatment response, complementing clinical observation of contingency/repair latency and habituation to predictable cues. Together, these elements yield a mechanism-informed formulation, specifying how caregiver–child contingency, intersubjective timing, and sensory gating jointly drive participation, learning, and well-being.

### 4.2. Psychotherapy: Adapting the Setting to Sensory and Attachment Needs

An attachment-informed, neurodiversity-affirming stance prioritizes felt safety, predictability, and graded engagement, calibrated to the child’s sensory/attentional profile.

(i) Optimize the therapeutic ecology. Configure spaces to minimize auditory/visual load, use predictable routines and graded proximity, and leverage interest-based materials to elicit approach without exceeding thresholds. Seating, lighting, and session pacing are used to facilitate micro-synchrony and repair, rather than expecting the child to conform to a standard clinic ecology.
(ii) Coach caregivers for co-regulation and mentalizing under stress. Randomized trials support parent training for disruptive behaviors in autistic children [73]; subsequent work documents gains in adaptive functioning when family-focused behavior management is sustained and generalized [74]. Caregiver-mediated strategies increase joint engagement in toddlers and improve communication-related outcomes when the coaching explicitly targets moment-to-moment contingency and repair [75,76]. Attachment-based programs (e.g., Attachment and Biobehavioral Catch-Up) enhance organization and stress regulation in high-risk dyads and can be sensory-titrated to preserve tolerable arousal [63]. Parent-mediated communication approaches (e.g., PACT) refine parental responsiveness to child communicative acts and arousal signals, thereby increasing opportunities for synchrony and naturalistic learning [64]. Building caregiver reflective functioning (see PRFQ above) via video-feedback and in-session narration helps parents label mental states during and after micro-mismatch/repair sequences without increasing child burden.
(iii) Mechanism-tracking and personalization. Use brief probes (gaze/prosody, paced touch, and predictable transitions) to identify signals that improve engagement; monitor HRV or recovery-time markers after brief, planned challenges; and adapt dosage for children high in environmental susceptibility/vantage sensitivity, who are predicted to show larger benefits in optimized sensory ecologies and may be disproportionately affected by chaotic settings [59,60,61]. Where sensory-based therapies are deployed, ensure protocol fidelity (e.g., Ayres Sensory Integration—SI) and tie goals to participation outcomes (e.g., communication/generalization in naturalistic settings) [62,71].

### 4.3. Education: Training Teachers and Caregivers to Read Relational Signals

Teacher-facing guidance (core, observable practices). Five micro-skills consistently support neurodivergent learners: (1) announce transitions (“pause–predict–prepare–proceed”) and use visual timers; (2) simplify and repeat key instructions with stable wording and pacing; (3) enact immediate micro-repairs after mismatches (name the emotion, restate the goal, offer an alternative); (4) use calm, rhythmic prosody and brief movement breaks to restore regulation; (5) leverage special interests as entry points for joint attention and task engagement.

Sensory-aware classroom ecology. Reduce auditory/visual competition (noise dampening, visual decluttering, preferential seating), provide predictable routines and micro-choices (first–then boards), and create a low-load corner for brief recovery. Individualized sensory plans should specify triggers, graded accommodations, and repair routines shared across staff and family.

Outcomes and monitoring. Prioritize participation and engagement (on-task joint work, number of successful repairs, and reduced arousal spikes) over mere symptom counts; track two or three indicators across the week and adjust accommodations accordingly. These practices are broadly applicable and can be implemented without specialized equipment, while more advanced options (e.g., fidelity-anchored sensory integration or physiological monitoring) can be considered when resources and training permit.

(a) Universal relational pedagogy. Teacher–child relationship quality predicts long-term academic and behavioral trajectories; professional learning enhances attunement and timely repair; predictable routines provide a relational scaffold for neurodivergent learners [77]. School-wide social-emotional learning (SEL) initiatives improve behavior and achievement at scale, fostering classrooms that are more conducive to co-regulation and inclusion [78].
(b) Sensory-aware classroom design. Proactive adjustments—noise management, seating, visual clarity, movement breaks, transition warnings—reduce prediction error and arousal spikes, particularly for students with SOR. Individualized sensory plans should specify triggers, accommodations, and repair routines (“pause–predict–prepare–proceed”), aligning classroom affordances with each child’s thresholds and attentional style (see Section 4.1).
(c) Family-centered goals and the ‘F-words’. Align educational/clinical targets with the ICF-derived ‘F-words’ (Function, Family, Fitness, Fun, Friends, Future), privileging participation, autonomy, and quality of life—key aims in neurodiversity-affirming care [79]. This shift moves beyond symptom reduction to contextual enablement: co-constructing co-regulatory niches across home and school that fit interests, sensory thresholds, and attentional styles while supporting learning. A synthesis of attachment-informed assessment and intervention priorities across ASD, ADHD, and prominent sensory differences is reported in Table 1.

### 4.4. Guidance for Caregivers and Youth (Plain-Language Practice Points)

- Keep routines predictable; preview changes early with simple visuals.
- Lower sensory load first (sound, light, visual clutter) before adding demands.
- Use special interests to invite shared attention and learning.
- When tension rises, stop, breathe, name the feeling, and try a small adjustment.
- Offer short, rhythmic breaks (walk, stretch, weighted pressure) to reset arousal.
- Teach a simple help-signal the child can use without words.
- Write a one-page sensory plan (triggers, what helps, what to avoid) and share it with the class.
- Celebrate tiny steps in participation, not only “perfect behavior”.
- Track what worked today (two lines in a diary); repeat the best elements tomorrow.
- Ask for professional support if distress persists, daily routines break down, or safety is a concern.

## 5. Future Research Directions

Advancing an attachment-informed account of neurodivergence requires longitudinal, mechanistic, and multimodal designs that can disentangle within-person developmental change from between-person selection, link caregiver–child processes to neural and physiological markers, and integrate laboratory with real-world measurement.

Longitudinal designs: Future work should estimate within-person cross-lagged effects while controlling for stable trait variance, e.g., random-intercept cross-lagged panel models (RI-CLPMs) to avoid the well-known conflation of between- and within-level dynamics in traditional CLPMs [80]. Combining RI-CLPM with dynamic structural equation modeling (DSEM) will permit modeling of micro-fluctuations in dyadic contingency and arousal (e.g., momentary synchrony, repair latency) and test how these fluctuations accumulate into change in affect regulation, intersubjectivity, and sensory responsivity [81]. Because the present framework predicts moderated mediation—attachment-related processes improving outcomes via affective, intersubjective, and sensory pathways, conditional on susceptibility/vantage sensitivity—analytic plans should preregister first- and second-stage moderation and probe conditional indirect effects with appropriate inference procedures [82].

**Neuroimaging + behavioral paradigms**. Example 1: Second-person; feasibility-first. In toddler–caregiver dyads, manipulate ostensive cues (gaze/prosody) within a brief play routine while titrating sensory load (predictable vs. unpredictable auditory/visual inputs). Record dual-EEG or fNIRS to index inter-brain coupling and collect HRV as a proximal allostatic marker. Hypothesis: Enhanced ostension under low sensory load increases coupling and shortens recovery times following perturbations. Example 2: Classroom titration; pragmatic. In an inclusive classroom, implement a micro-randomized schedule of noise-reduction and transition warnings (e.g., five min pre-alerts). Outcomes: Within-student changes in EMA (ecological momentary assessment) ratings of arousal/engagement, teacher-coded micro-repairs, and participation. Hypothesis: Sensory containment and predictable transitions reduce arousal spikes and increase joint engagement.

Mechanistic studies should adopt second-person neuroscience to preserve the bidirectionality and timing of caregiver–child exchanges during measurement (e.g., dual-EEG/fNIRS hyperscanning during naturalistic play, still-face perturbations, or graded sensory challenges), indexing inter-brain coupling as a mediator between attachment signals (gaze, prosody, touch), and regulatory outcomes [83]. A growing body of parent–child fNIRS work supports the feasibility and construct validity of these designs for capturing frontal systems engaged in co-regulation [84]. Parallel circuit-level probes—for instance, fMRI or MEG assays of thalamocortical modulation and multisensory integration—should be aligned to SOR phenotypes to test whether reductions in prediction error under attachment-consistent scaffolding correspond to normalization of gating/connectivity patterns [85].

**Multimodal integration (behavior, neurophysiology, questionnaires).** A critical step is to fuse behavioral micro-coding (contingency, repair), physiology (HRV, EDA, cortisol), brain signals (EEG/fNIRS/fMRI), and validated questionnaires (e.g., social responsivity; parental reflective functioning) within unified analytic frameworks spanning the clinic, home, and school. EMA combined with wearables can quantify the everyday ecology of co-regulation and the sensory load that shapes participation [86]. HRV is a tractable vagal regulation biomarker and can serve as a proximal outcome and titration guide for sensory-aware interventions when acquisition/reporting follow psychophysiological best-practice recommendations [87]. On the brain side, multimodal data-fusion approaches (e.g., joint ICA, linked matrix factorization) can reveal latent dimensions that cut across imaging modalities and map onto clinical/behavioral phenotypes, accelerating target identification for personalization [88]. Complementary hyperscanning (e.g., NIRS-based dual-brain recordings) improves ecological validity for dyadic tasks and can localize fast synchrony-related processes within broader cortical networks [89]. To boost power and reproducibility, teams should leverage open resources (e.g., ABIDE for ASD neuroimaging, extended with attachment/sensory phenotypes in new cohorts) and adhere to open-science norms (preregistration, registered reports, transparent pipelines, and data/code sharing) [90,91].

**A phased translational agenda**. I envisage a pipeline that (i) establishes measurement models for attachment-relevant processes under varying sensory loads (years 1–2); (ii) tests causal mechanisms via micro-randomized or sequential multiple assignment trials that manipulate caregiver signals and sensory context (years 2–4); and (iii) implements pragmatic, school-embedded trials assessing participation and quality-of-life outcomes (years 4–6). Throughout, analyses should center moderated mediation and individual differences in susceptibility, enabling precise tailoring of alliance design, caregiver coaching, and classroom accommodations. Integrating longitudinal, second-person, and multimodal methods can move the field from correlational description to a causal, mechanistic, and clinically actionable science consistent with a neurodiversity-affirming and attachment-informed paradigm.

**Prioritization and Feasibility**.

**Tier 1 (immediate, practice-proximal).** Use brief repair micro-coding in play/classroom samples, short emotional-availability observations, and structured sensory checklists, optionally add light-touch HRV. Track participation-relevant outcomes (joint engagement, successful repairs, arousal spikes) to inform accommodations.

**Tier 2 (translational).** Deploy dual-EEG/fNIRS hyperscanning in naturalistic interactions with controlled manipulations of ostension (gaze, prosody) and sensory load; test whether inter-brain coupling mediates gains in engagement when sensory environments are titrated.

**Tier 3 (advanced).** Apply RI-CLPM/DSEM to intensive longitudinal cohorts to separate within- from between-person effects and to model sensitive periods. Combine psychophysics of habituation/prediction error with fMRI/MEG assays of thalamocortical gating and multimodal data fusion to derive targets for precision supports; preregister analyses and share data/code.

These examples align with a staged agenda: Tier 1—practice-proximal probes (micro-coding, HRV, sensory checklists), Tier 2—translational second-person paradigms (dual-EEG/fNIRS), and Tier 3—intensive longitudinal and circuit-level studies (RI-CLPM, DSEM; fMRI/MEG).

**Mapping hypotheses to tests.**

**H1:**  *Caregiver contingency → faster recovery/top–down control. Test via micro-coded repair + stress-recovery indices (behavioral or HRV); criterion: shorter recovery constants after perturbations.*

**H2:**  *Synchrony → joint engagement/mentalizing. Test via dual-EEG/fNIRS with ostensive manipulations; criterion: increased inter-brain coupling and joint engagement under titrated load.*

**H3:**  *Sensory-aware co-regulation → reduced SOR reactivity. Test via classroom titration and sensory-integration protocols; criterion: fewer overload episodes, better participation.*

**H4:**  *Susceptibility moderates benefits. Test via baseline susceptibility markers (sensory thresholds, vagal tone); criterion: steeper gains for high-susceptibility subgroups and dose–response patterns.*

Beyond dyadic mechanisms, the broader post-digital sociocultural ecology likely modulates co-regulation and the reading of relational signals. Contemporary analyses of subjectivation argue that ubiquitous digital mediation can fragment autobiographical self-continuity and intensify reliance on external validation, with potential downstream effects on mentalization and attachment signaling—especially in youth cohorts for whom online sociality is pervasive. Integrating this macro-level lens with the present framework suggests that attachment-informed supports may need to explicitly scaffold identity coherence and digital hygiene (e.g., predictable off-screen co-regulatory routines, and narration of online/offline experiences) to sustain felt security under heightened sensory and attentional load. This perspective motivates future work testing whether digital-context stressors moderate the pathways from caregiver contingency to affect regulation, intersubjectivity, and sensory gating outlined here [92].

## 6. Conclusions

This article advanced an attachment-informed, neurodiversity-affirming account of neurodivergence, positioning early attachment experiences as developmentally upstream organizers of three partially overlapping systems—affect regulation, intersubjectivity, and sensory processing—that channel trajectories toward distinct behavioral phenotypes. Converging evidence from synchrony science and affective neuroscience indicates that sensitive, contingent caregiving scaffolds allostatic regulation and prefrontal control over limbic salience, with downstream benefits for joint engagement and mentalizing under affective load. In parallel, sensory research implicates prediction error dynamics and thalamocortical modulation in autistic SOR; titrating sensory ecology within relationships can reduce arousal, sustain engagement, and improve participation. Taken together, a transactional view emerges: neurodivergent dispositions shape how attachment signals are expressed and used, while attachment processes recalibrate regulatory systems over time.

Clinically, the framework reorients formulation and care around mechanisms rather than labels. Assessment couples standard diagnostic tools with measures of dyadic contingency/repair, caregiver reflective functioning, and sensory thresholds, optionally complemented by physiological indices (e.g., HRV) to index allostatic load. Intervention becomes the co-construction of felt security under tolerable arousal, achieved through alliance design (predictable, low-load settings), caregiver coaching for moment-to-moment co-regulation, and fidelity-anchored sensory supports. Because children differ in environmental susceptibility/vantage sensitivity, personalization is principled: those at the tails of susceptibility distributions are expected to show the largest gains when contexts are enriched and calibrated.

Theoretically, the proposal bridges attachment constructs (security/disorganization; sensitivity/synchrony; mentalizing under stress) with neurodevelopmental mechanisms (attention, motivation, sensory gating), moving beyond siloed accounts by specifying testable pathways and bidirectional feedback. Methodologically, the next phase should prioritize longitudinal, mechanistic, and multimodal designs, e.g., RI-CLPM/DSEM to separate within- from between-person effects, second-person hyperscanning to assay inter-brain coupling under graded sensory load, and data-fusion pipelines aligning behavioral micro-processes, physiology, and neuroimaging. In tandem with open resources and open-science practices, such designs can accelerate the transition from correlational studies to causal, clinically actionable knowledge.

In conclusion, viewing neurodivergence through an attachment-developmental lens clarifies how affect regulation, intersubjectivity, and sensory processing co-determine trajectories. The framework yields testable pathways and actionable levers: calibrating the sensory ecology to sustain felt safety; coaching caregivers in micro-repair and reflective functioning; and personalizing supports by environmental susceptibility. These targets are measurable (e.g., HRV; micro-coding of repair; dual-brain coupling) and malleable, offering a pragmatic route to improved participation, autonomy, and well-being in autistic and ADHD populations.

## Figures and Tables

**Table 1:** Attachment-informed clinical implications by neurodivergent profile.

Profile	Assessment (Add-Ons to Standard Tools)	Psychotherapy Focus	School Strategies	Key References
ASD	Core: Include EA observation and PRFQ screening to capture relational functioning; sensory profile for modulation thresholds. Specialized: Incorporate HRV or stress biomarkers during structured observation.	Core: Parent-mediated synchrony training (PACT principles), repair coaching, and predictable setting. Specialized: Integrate sensory modulation strategies with social communication interventions.	Core: Structured routines, visual supports, and pre-transition cues. Specialized: Collaborative sensory plan with occupational therapist, adaptive seating, and sensory corners.	[57,64,69,70,71,72,73,74,75,76]
ADHD	Core: Emotional availability coding, PRFQ, and multi-informant rating scales. Specialized: Incorporate brief executive function probes and attention fluctuation mapping	Core: Parent coaching to enhance contingency and repair; behavioral activation with co-regulation focus. Specialized: HRV biofeedback for arousal regulation and adaptive pacing interventions.	Core: Predictable classroom routines, structured transitions, interest-based reinforcement. Specialized: Teacher training on recognizing micro-signals of overload and recovery strategies	[30,31,66,67,68]
Prominent Sensory Differences (SOR/modulation)	Core: Sensory profile assessment; structured observation of arousal to graded stimuli. Specialized: HRV, electrodermal activity (EDA), and thalamocortical modulation indices if available.	Core: Graded exposure and co-regulation under predictable conditions; parent coaching on titration. Specialized: Fidelity-based SI program integration and sensory-based relational therapy.	Core: Noise management, visual clarity, movement breaks. Specialized: Sensory zoning plans and environmental adaptations individualized to threshold maps.	[14,26,32,33,34,71,72]

**Legend**: Early attachment experience → Affect regulation/intersubjectivity/sensory processing → neurodivergent phenotype. **Edges:** Solid arrows denote hypothesized directional influence; double-headed arrows denote bidirectional coupling. **Levels:** Microtemporal (dyadic synchrony and repair), developmental (consolidation of regulatory capacities), and contextual (environmental load and accommodations). Legend. ASD = Autism spectrum disorder. ADHD = Attention-deficit/hyperactivity disorder. SOR = Sensory over-responsivity. EA = Emotional availability. PRFQ = Parental Reflective Functioning Questionnaire. HRV = Heart Rate Variability. SI = Ayres Sensory Integration. Key references refer to the manuscript’s numerical reference list.

## Data Availability

No new data were created or analyzed in this study. Data sharing is not applicable to this article.

## Abbreviations

The following abbreviations are used in this manuscript:

ASD	Autism Spectrum Disorder
ADHD	Attention-Deficit/Hyperactivity Disorder
DSEM	Dynamic Structural Equation Modeling
EA	Emotional Availability
EMA	Ecological Momentary Assessment
fNIRS	Functional Near-Infrared Spectroscopy
fMRI	Functional Magnetic Resonance Imaging
HRV	Heart-rate variability
MEG	Magnetoencephalography
PRFQ	Parental Reflective Functioning Questionnaire
RI-CLPM	Random-Intercept Cross-Lagged Panel Model
SI	Ayres Sensory Integration
SOR	Sensory Over-Responsivity
